# Supplementary material for: Challenges Facing Two Outbreaks of Carbapenem-Resistant Acinetobacter baumannii: From Cefiderocol Susceptibility Testing to the Emergence of Cefiderocol-Resistant Mutants
Source: Antibiotics (Basel). 2024 Aug 21;13(8):784. doi: 10.3390/antibiotics13080784 (PMC11350900; doi:10.3390/antibiotics13080784)
Supplement: Supplementary file 1 [file antibiotics-13-00784-s001.zip › antibiotics-3150155-supplementary.pdf]

Table S1. Source of infection, etiology, cefiderocol susceptibility test according EUCAST criteria, targeted treatment, and outcomes in patients infected by CRAB during both outbreaks (from October 2022 to November 2023).

	Reason for admission	CRAB Infection	Polymicrobial	FDC disk diffusion	FDC MIC (mg/L)	Targeted treatment	Outcome CRAB infection	Recurrent infection	Source infection	FDC disk diffusion	FDC MIC (mg/L)	Treatment	Outcome
1	Burn	BSIc	*P. aeruginosa*	S	0.5	FDC	CC	YES	BSI$_2$	**R**	2	COL	Alive
2	Burn	BSI$_2$	*Proteus*	S	0.5	COL + PIPTAZ	CC						Deceased
3	Burn	BSI2		S		FDC	CC						Alive
4	Medical	VAP	*P. aeruginosa*	S	1	COL +MRP	TF	YES	VAP	NA		CFD	Deceased
5	Burn	BSI$_1$	*S. aureus*	S	1	COL + LIN	CC						Alive
6	Trauma	VAP		S		FDC+ COL	CC						Alive
7	Burn	BSI$_2$	*K. pneumoniae*$^{oxa\text{-}48}$	S	0.5	FDC	CC						Deceased
8	Medical	VAP		S		FDC	TF	YES	VAP	NA		CFD	Deceased
9	Burn	BSI$_2$	*S. aureus*	S		FDC + COL+ LIN	CC						Alive
10	Burn	VAP		S		--- *	---						Deceased
11	Burn	VAP		S	1	FDC + LIN	CC	YES	BSI$_2$	S	1	CFD	Deceased
12	Burn	BSI$_C$		S		FDC	CC						Alive
13	Burn	VAP		S	1	FDC	CC						Alive
14	TEN	VAP	*Aspergillus* spp	**R**	2 S	COL + SUL	TF	YES	VAP	**R**	**4 R**	COL	Deceased
15	Medical	VAP		S		FDC + COL	CC						Alive
16	Burn	VAP	*S. marcenses*	S		FDC	CC						Alive
17	Burn	VAP		**R**	2 S	COL+ SULB	CC						Alive
18	TEN	BSI$_1$	*P. aeruginosa*	**R**	2 S	COL	CC						Alive
19	Burn	BSI$_C$		**R**	**16 R**	FDC+ COL + SUL*	CC						Alive
20	Burn	BSI$_C$	*P. aeruginosa*^{VIM}	S		FDC	CC						Alive

TEN: toxic epidermal necrolysis; BSI: bloodstream infections (c: catheter related, 2: secondary BSI, 1: unknown BSI); VAP: ventilator-associated pneumonia; FDC: cefiderocol; COL: colistin; SUL: sulbactam; MRP: meropenem; PIPTAZ: Piperacillin/Tazobactam; LIN: Linezolid; CC: Clinical cure; TF: treatment failure; NA: not available; *10 case: not received treatment because decision to withhold life-sustaining treatment do to severity burn injury. Case 19 and 20 belongs to the second outbreak. *19 case: synergy FDC + SUL was found *in vitro* test.
